# Cortical Gyrification and Sulcal Spans in Early Stage Alzheimer's Disease

**DOI:** 10.1371/journal.pone.0031083

**Published:** 2012-02-21

**Authors:** Tao Liu, Darren M. Lipnicki, Wanlin Zhu, Dacheng Tao, Chengqi Zhang, Yue Cui, Jesse S. Jin, Perminder S. Sachdev, Wei Wen

**Affiliations:** 1 Centre for Quantum Computation and Intelligent System, Faculty of Engineering and Information Technology, University of Technology Sydney, New South Wales, Australia; 2 Brain and Ageing Research Program, School of Psychiatry, University of New South Wales, Sydney, New South Wales, Australia; 3 Neuropsychiatric Institute, Prince of Wales Hospital, Randwick, New South Wales, Australia; 4 School of Design, Communication & I.T., The University of Newcastle, New South Wales, Australia; Nathan Kline Institute and New York University School of Medicine, United States of America

## Abstract

Alzheimer's disease (AD) is characterized by an insidious onset of progressive cerebral atrophy and cognitive decline. Previous research suggests that cortical folding and sulcal width are associated with cognitive function in elderly individuals, and the aim of the present study was to investigate these morphological measures in patients with AD. The sample contained 161 participants, comprising 80 normal controls, 57 patients with very mild AD, and 24 patients with mild AD. From 3D T1-weighted brain scans, automated methods were used to calculate an index of global cortex gyrification and the width of five individual sulci: superior frontal, intra-parietal, superior temporal, central, and Sylvian fissure. We found that global cortex gyrification decreased with increasing severity of AD, and that the width of all individual sulci investigated other than the intra-parietal sulcus was greater in patients with mild AD than in controls. We also found that cognitive functioning, as assessed by Mini-Mental State Examination (MMSE) scores, decreased as global cortex gyrification decreased. MMSE scores also decreased in association with a widening of all individual sulci investigated other than the intra-parietal sulcus. The results suggest that abnormalities of global cortex gyrification and regional sulcal span are characteristic of patients with even very mild AD, and could thus facilitate the early diagnosis of this condition.

## Introduction

As the most common cause of dementia, Alzheimer's disease (AD) is characterized by an insidious onset of cerebral atrophy and progressive cognitive decline [1], [2]. Brain regions showing abnormalities in AD, including the paralimbic and heteromodal association areas, have been well documented using postmortem brain tissue [1], [3], [4]. More recently, magnetic resonance imaging (MRI) has been used to quantitatively study the neuroanatomic abnormalities of individuals with AD *in vivo*. MRI-based investigations have utilized volumetric measures of either regions of interest (ROIs) [5], [6], [7] or the whole brain [8], [9], [10], voxel-based morphometry (VBM) [11], [12], [13], [14], [15], and cortical thickness [16], [17]. Across these studies, AD has often been associated with atrophy or cortical thinning in a number of brain regions, including frontal [7], [12], [17], temporal [7], [11], [12], [17], parietal [7], [17] and hippocampal [6], [7], [11], [12], [18].

Variations of cortical folding morphology offer another approach to investigating neuroanatomic differences, and have received recent interest following the development of sophisticated 3D-based image-processing techniques [19]. Sulcal folds are the principal surface landmarks of the human cerebral cortex, and exhibit structurally complex patterns [20] that are postulated to reflect underlying connectivity [21]. Indeed, cortical folding patterns have been used to predict cytoarchitecture [22]. In addition to being a macroscopic probe for hidden architectural organization, folding geometry may also provide information on developmental events [19]. Recent studies have identified morphological differences in the sulci of some professional groups, including musicians [23], patients with psychiatric and neurological conditions like schizophrenia [24] and bipolar disorder [25], and individuals with cerebral small vessel disease [26], [27]. Changes of folding geometry have also been shown to develop with aging [28], [29] and be associated with cognitive decline [30]. The sulcal patterns associated with mild cognitive impairment (MCI) and AD have also been investigated, with Im et al. [31] finding that the sulci of individuals with either of these conditions had less curvature (reflecting greater widening) and depth than those of cognitively normal controls. These differences were observed to be the largest in the temporal lobe.

The present study aimed to investigate cortical folding and sulcal spans in AD, and expanded upon previous research in several important ways. First, global measurements of cortical gyrification were obtained using the global sulcal index (g-SI). This is a new index whereby a larger g-SI reflects a greater degree of global cortex gyrification or folding [24]. Our group previously found that g-SI not only decreases with age [29], but also with cognitive decline in the elderly [30]. We therefore hypothesized for the present study that individuals with AD would have a lower g-SI than cognitively normal controls. Second, the earlier study by Im et al. [31] investigated sulcal morphology on the level of brain lobes. Our investigation was more fine-grained than this, with analyses conducted at the level of five individual sulci. We have previously found the spans of these sulci to be correlated with age [29] and cognitive function [30] in the elderly. Third, we explored how Mini-Mental State Examination (MMSE) scores were related to cortical folding and sulcal spans. The MMSE is widely used in both clinical and research settings to assess patients with AD [32], [33], but associations between its scores and either g-SI or individual sulcal spans do not appear to have been investigated. Fourth, previous studies were reported on patients with a severity of AD ranging from mild to moderate. In the present study, we focused specifically on even earlier stages of AD, including the very mild. Understanding the brain changes associated with AD at its mildest stage may facilitate the development of early interventions for this debilitating condition.

## Methods

### Ethics Statement

For the purpose of this analysis we used OASIS subject data that was previously collected under several study protocols at Washington University. All studies were approved by the University's Institutional Review Board (IRB). All subjects gave written informed consent at the time of study participation. The University's IRB also provided explicit approval for open sharing of the anonymized data.

### Participants

Participants were drawn from the Open Access Series of Imaging Studies (OASIS) database (http://www.oasis-brains.org) [34]. Our investigation was restricted to right-handed participants who were at least 62 years old, the youngest age at which any individual in the OASIS database is classified as having early-stage AD. Of 193 potential participants, 32 were excluded due to failures in imaging processing steps that included skull stripping (n = 5), segmentation (n = 6) and sulcal recognition (n = 21). The remaining individuals were classified on the basis of Clinical Dementia Rating scale (CDR) [35], [36] scores as having normal cognition (CDR = 0, n = 80), very mild AD (CDR = 0.5, n = 57) or mild AD (CDR = 1, n = 24). The age, sex ratio, estimated total intracranial volume (eTIV), and MMSE score for individuals in each of these groups were obtained directly from the OASIS database.

### Image acquisition

For each participant, we obtained from the OASIS database a single image with a high contrast-to-noise ratio. As described by Marcus et al. [34], these images were produced by averaging across 3 or 4 motion-corrected T1-weighted magnetization prepared rapid gradient-echo (MP-RAGE) images, which were acquired on a 1.5T Vision scanner (Siemens, Erlangen, Germany) within a single session during which cushioning and a thermoplastic face mask were used to minimize head movements. The MP-RAGE parameters were empirically optimized for gray-white contrast, with repetition time = 9.7 ms, echo time = 4.0 ms, inversion time = 20 ms, delay time = 200 ms, flip angle = 10°, orientation = sagittal, resolution = 256×256 matrix, slices = 128, and thickness = 1.25 mm.

### Image pre-processing

Cortical sulci were extracted from images via the following three steps. First, we removed non-brain tissue to produce images containing gray matter (GM), white matter (WM) and cerebrospinal fluid (CSF). This was done by warping a brain mask defined in the standard space back to the raw T1-weighted structural MRI scan. The brain mask was obtained with an automated skull stripping procedure based on the SPM5 skull-cleanup tool [37]. Second, we segmented images into GM, WM and CSF using a fuzzy-classifier-based, anatomical segmentation method, after applying a field inhomogeneity bias correction [38]. Third, individual sulci were identified and extracted using the BrainVisa (BV) sulcal identification pipeline (version 3.2.0; http://brainvisa.info/). The medial surface of the cortical folds was calculated using a homotopic erosion technique [39] and a crevasse detector was used to reconstruct sulcal structure as the medial surface from the two opposing gyral banks that spanned from the most internal point of the sulcal fold to the convex hull of the cortex [38]. A sulcal labeling tool incorporating 500 artificial neural network-based pattern classifiers [40] was used to label sulci. Sulci that were mislabeled by BV were manually corrected.

### Morphological measures

**Figure 1:**
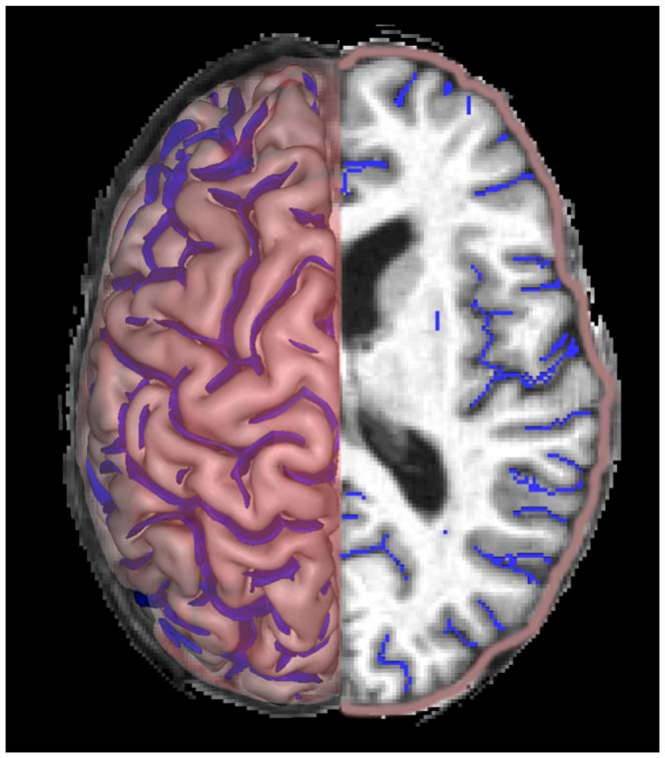
The global sulcal index (g-SI) for each hemisphere represents the ratio between the total sulcal area (blue) and the outer cortical area (red).

For each hemisphere, we calculated g-SI as the ratio between total sulcal area and outer cortical area [24], [25] (see Figure 1). We calculated g-SI automatically, with no manual intervention using BV.

**Figure 2:**
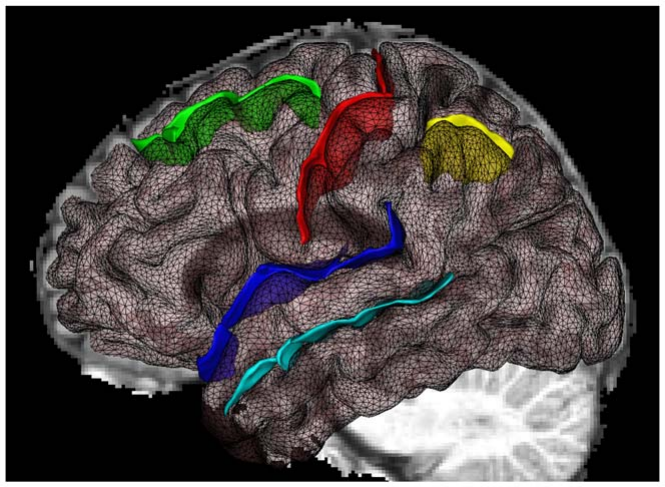
The five sulci measured were: superior frontal sulcus (green), central sulcus (red), Sylvian fissure (dark blue), superior temporal sulcus (light blue), and intra-parietal sulcus (yellow).

Also for each hemisphere, we determined the average sulcal span for each of five sulci: superior frontal, intra-parietal, superior temporal, central, and Sylvian fissure (see Figure 2). Sulcal span was defined as the average 3D distance between opposing gyral banks along the normal projections to the medial sulcal mesh [41], [42].The five sulci investigated in the present study were chosen because they are present in all individuals, large and relatively easy to identify (facilitating error detection), and located on different cerebral lobes.

In order to help understand the basis of any group differences in cortical gyrification or sulcal spans, we also calculated a measure of cortical thickness. This was done using FreeSurfer 3.0.5 (http://surfer.nmr.mgh.harvard.edu/) in a process that involved automated Talairach transformation, segmentation of the subcortical WM and deep GM volumetric structures [43], intensity normalization, tessellation of the GM/WM boundary, automated correction of topology defects, surface deformation to form the GM/WM boundary and GM/CSF boundary [44]. Cortical thickness was calculated at each vertex [43], and averaged over the whole cortex.

### Statistical analysis

Differences between the normal, very mild AD, and mild AD groups were investigated using ANOVA for age, MMSE score, and a chi-square test for sex ratio. Group differences were first investigated with ANCOVA for g-SI and average cortical thickness, and with MANCOVA for the five sulcal spans. Significant main effects for MMSE score, g-SI, average cortical thickness and individual sulcal spans were followed-up using Bonferroni post-hoc tests. All g-SI, average cortical thickness and sulcal span comparisons were adjusted for age, sex and eTIV. Associations between both g-SI and sulcal spans and MMSE scores were explored using partial correlations controlling for age, sex and eTIV. We initially conducted all analyses with hemisphere as a factor, but there were no differences between the left and right hemispheres and the results reported are for values averaged across these. Analyses were performed using SPSS (version 18.0.0) and *p*<0.05 considered statistically significant.

## Results

Table 1 shows the mean age, sex, eTIV, MMSE, average cortical thickness, g-SI and sulcal spans of the normal, very mild AD and mild AD groups. There were no significant group differences in age, sex or eTIV, but the mean MMSE score of the normal group was significantly higher than that of the very mild AD group, which was in turn significantly higher than that of the mild AD group. Average cortical thickness was lower in both the very mild and mild AD groups than in the normal group, though no different between AD groups. In terms of g-SI, there was a progressive decline from the normal group to the very mild AD group and from the very mild AD group to the AD group. An overall significant MANCOVA result indicated group differences in sulcal spans (F_(4,156)_ = 3.013, *p* = 0.020), which follow-up comparisons identified as mostly reflecting wider sulcal spans in the mild AD group than in the normal group. This was the case for the Sylvian fissure and the central, superior frontal and superior temporal sulci (Table 1). The span of the superior frontal sulcus was also greater in the mild AD group than in the very mild AD group. The only other significant difference found was a greater span of the Sylvian fissure in the very mild AD group than in the normal group. The span of the intra-parietal sulcus did not differ between any of the groups.

**Table 1:** Demographic characteristics and morphological measure values of the normal, very mild AD, and mild AD groups.

Characteristic/Measure	Normal(n = 80)	Very mild AD(n = 57)	Mild AD(n = 24)	F^a^	*p* a
Age, y	76.2±8.1	75.8±7.3	77.3±7.3	0.34	0.716
Males (%)	27.5	43.9	25.0	4.83^b^	0.089
eTIV, ml	1452±152	1486±178	1469±119	0.77	0.463
Average cortical thickness, mm	2.19±0.08	2.13±0.09^c^	2.09±0.11^c^	11.89	<0.001
MMSE score	28.9±1.3	25.4±3.7^c^	21.2±3.5^c^^,^ d	80.48	<0.001
g-SI	1.30±0.16	1.22±0.16^c^	1.10±0.14^c^^,^ d	17.13	<0.001
Sulcal span, mm					
Sylvian fissure	5.56±0.91	5.98±0.93^c^	6.37±0.91^c^	9.62	<0.001
Intra-parietal	4.35±0.81	4.34±0.63	4.61±0.44	1.18	0.310
Central	3.54±0.63	3.63±0.72	3.92±0.78^c^	3.04	0.051
Superior frontal	4.40±0.59	4.43±0.58	4.84±0.66^c^^,^ d	5.00	0.008
Superior temporal	3.99±0.65	4.12±0.70	4.45±0.65^c^	5.49	0.005

Values are mean ± SD unless otherwise indicated.

a Results of omnibus test across all three groups.

b Chi-square statistic.

c Significant difference from Normal with post-hoc Bonferonni test (*p*<0.05).

d Significant difference from Very mild AD with post-hoc Bonferonni test (*p*<0.05).

Relationships between MMSE scores and each of sulcal spans and global cortex gyrification are shown in Table 2. There was a significant positive correlation between g-SI and MMSE score. Significant negative correlations between MMSE score and sulcal span were found for the Sylvian fissure, central, superior frontal and superior temporal sulci. The association between MMSE score and intra-parietal sulcus span did not reach the level of statistical significance.

**Table 2:** Associations between morphological measures and MMSE scores.

Measure	Partial r	*p*
g-SI	0.424	<0.001
Sulcal span		
Sylvian fissure	−0.263	<0.001
Intra-parietal	−0.151	0.058
Central	−0.180	0.024
Superior frontal	−0.263	<0.001
Superior temporal	−0.306	<0.001

## Discussion

The present study investigated cortical gyrification and sulcal spans in early stage AD. Compared to cognitively normal controls, we found that individuals with early stage AD had lower levels of global cortex gyrification, as measured by g-SI. This is consistent with a recent report of patients with mild AD having a lower gyrification index value than normal controls [45]. Extending upon these results, we also found a greater reduction in global cortex gyrification for individuals with mild AD than for those with very mild AD. This difference in gyrification reflects an increase in clinical symptom severity from very mild AD to mild AD [16], and suggests a potential role for g-SI in tracking progression of the disease. We also conducted a focused investigation of five particular sulci, and found the width of four of these to be greater in individuals with mild AD than in cognitively normal controls. AD is characterized by progressive cognitive decline [1], [2], and the results of the present study are thus consistent with cognitive decline in the elderly being associated with decreases in cortical folding and increases in sulcal span [30].

A previous study found sulcal widening in individuals with AD on the level of brain lobes within each of the frontal, parietal, temporal and occipital lobes [31]. The present study extends this work by finding AD-associated widening of individual sulci within brain lobes, particularly the superior frontal and superior temporal sulci. We found no inter-group differences in the width of the intra-parietal sulcus, suggesting that it may not have been a major contributor to the parietal lobe effects reported previously [31] or to the reduction in global cortex gyrification we report here. Indeed, our findings of AD-associated widening specifically of the superior frontal and superior temporal sulci are consistent with the spatial distribution of neurodegenerative change in AD reported by post-mortem studies [1], [3]. They are also consistent with voxel-based morphometric [11], [12], [13], [14] and cortical thickness studies [16], [17], [46] in which GM atrophy was seen primarily in the frontal and temporal regions of individuals with mild AD. Given these findings, and the fact that it separates the frontal and temporal lobes, it is not surprising that the Sylvian fissure was the sulcus we found to exhibit the largest increase in width between individuals with normal cognition and those with mild AD. Also noteworthy is that the Sylvian fissure was the only sulcus for which width differed between individuals with normal cognition and those with very mild AD. This suggests that, alongside g-SI, width of the Sylvian fissure could be a useful neuroanatomical marker of early-stage AD.

The idea that GM atrophy underlies changes in sulcal width associated with AD is consistent with reductions in gyral GM volume being associated with increases in sulcal width [31], [47] and supported by our finding that average cortical thickness was lower in individuals with early stage AD than in normal controls. Even so, it is likely that WM alterations also have a role to play, given reports that sulcal width reflects the integrity of both GM and WM [27], [41]. Indeed, previous studies have found reductions in WM within a number of brain regions in patients with AD [48].

The differences in global cortex gyrification and sulcal spans between cognitively normal controls and participants with AD are complemented by the finding that MMSE scores were positively associated with g-SI and negatively associated with the span of each of four individual sulci (central, superior frontal, superior temporal, and Sylvian fissure). This supports our previous report of associations between cognitive test performance and cortical morphology at both the regional and global levels in elderly individuals [30], and is not unexpected given the capacity of the MMSE to assess AD severity [33]. Accordingly, there is consistency in our findings that the span of the intra-parietal sulcus neither differentiated between cognitively normal controls and participants with AD, nor had a statistically significant association with MMSE scores. However, our results do not exclude an involvement of the parietal lobe in the early stages of AD, which could involve metabolic changes more so than structural changes, or structural changes in parietal regions that minimally affect the intra-parietal sulcus [49].

The present study has a limitation in being cross-sectional, such that causal inferences between sulcal width and dementia severity cannot be made. An aim of future research should be to longitudinally track changes in g-SI and sulcal width within individuals who experience an increasing severity of AD, and to do so into the moderate and severe stages of AD that are beyond the very mild and mild levels that we report here.

We have demonstrated abnormalities of global cortex gyrification and regional sulcal spans in very mild and mild AD. The results of the present study suggest that such abnormalities are an important and salient characteristic of patients in the early stages of AD, and could thus potentially aid in the timely and accurate clinical diagnosis of this debilitating neurodegenerative condition.
